# Association of an MHC Class II Haplotype with Increased Risk of Polymyositis in Hungarian Vizsla Dogs

**DOI:** 10.1371/journal.pone.0056490

**Published:** 2013-02-14

**Authors:** Jonathan Massey, Simon Rothwell, Clare Rusbridge, Anna Tauro, Diane Addicott, Hector Chinoy, Robert G. Cooper, William E. R. Ollier, Lorna J. Kennedy

**Affiliations:** 1 Centre for Integrated Genomic Medical Research (CIGMR), Institute of Population Health, Faculty of Medical and Human Sciences, The University of Manchester, Manchester, United Kingdom; 2 Stone Lion Veterinary Hospital, London, United Kingdom; 3 Alcombe Veterinary Surgery, London, United Kingdom; 4 Hungarian Vizsla Breed Club, Royal Tunbridge Wells, United Kingdom; 5 Rheumatic Diseases Centre, Manchester Academic Health Science Centre, The 6 University of Manchester, Salford Royal NHS Foundation Trust, Salford, United Kingdom; National Institutes of Health, United States of America

## Abstract

A breed-specific polymyositis is frequently observed in the Hungarian Vizsla. Beneficial clinical response to immunosuppressive therapies has been demonstrated which points to an immune-mediated aetiology. Canine inflammatory myopathies share clinical and histological similarities with the human immune-mediated myopathies. As MHC class II associations have been reported in the human conditions we investigated whether an MHC class II association was present in the canine myopathy seen in this breed. 212 Hungarian Vizsla pedigree dogs were stratified both on disease status and degree of relatedness to an affected dog. This generated a group of 29 cases and 183 “graded” controls: 93 unaffected dogs with a first degree affected relative, 44 unaffected dogs with a second degree affected relative, and 46 unaffected dogs with no known affected relatives. Eleven DLA class II haplotypes were identified, of which, DLA-DRB1*02001/DQA1*00401/DQB1*01303, was at significantly raised frequency in cases compared to controls (OR = 1.92, p = 0.032). When only control dogs with no family history of the disease were compared to cases, the association was further strengthened (OR = 4.08, p = 0.00011). Additionally, a single copy of the risk haplotype was sufficient to increase disease risk, with the risk substantially increasing for homozygotes. There was a trend of increasing frequency of this haplotype with degree of relatedness, indicating low disease penetrance. These findings support the hypothesis of an immune-mediated aetiology for this canine myopathy and give credibility to potentially using the Hungarian Vizsla as a genetic model for comparative studies with human myositis.

## Introduction

A polymyositis (an immune-mediated form of generalised inflammatory myopathy) with specific characteristics is being increasingly observed in the Hungarian Vizsla dog [1], [2]. Affected dogs generally present with difficulty eating and drinking (dysphagia), regurgitation, and sialorrhea. Involvement of the masticatory muscle can result in marked masticatory muscle atrophy and generalised muscle involvement can lead to exercise intolerance and weakness. Definitive diagnosis is by muscle biopsy histopathology, which commonly shows endomysial and/or perimysial infiltration by lymphocytes and histiocytes [1]. In one study, using three dogs, CD4+ T-cells were found in excess of CD8+ T-cells which advocates a divergent aetiology to other cases of canine polymyositis [1]. Other diagnostic tests, such as serum creatine kinase concentration, electromyography, fluoroscopy of the oesophagus, and Magnetic Resonance Imaging (MRI) of affected muscle groups can aid in diagnosis. It is also important to rule out other neuromuscular diseases and infectious myopathies. Immunosuppressive drug therapies have been used to treat the disease and dogs appear to respond well [3]. This has furthered support for the hypothesis of an immune-mediated aetiology for myositis in this particular breed.

Canine immune-mediated inflammatory myopathies share many clinical and histological characteristics with those in humans [4]. The human immune-mediated myopathies are broadly separated into three sub-groups: polymyositis (PM), dermatomyositis (DM) and inclusion body myositis (IBM). There are known disease risk associations with all of these human conditions with allelic variants in the Human Leukocyte Antigen (HLA) class I and class II regions [5].

The incidence of polymyositis in the general canine population is unknown. It has been described at increased frequency in Boxers and Newfoundlands [6], although this is likely a specific form of the disease, with evidence of circulating sarcolemma autoantibodies [7]. Studies in canine polymyositis have shown increased expression of Dog Leukocyte Antigen (DLA) class I and class II molecules on the surface of muscle fibres and infiltrating cells [1] but no associated alleles or haplotypes have as yet been identified.

Other forms of inflammatory myopathies in dogs include masticatory muscle myositis, which appears to be unique to this species. Like in polymyositis, increased expression of DLA class I and II expression has been shown [8]. Dermatomyositis is an inflammatory disease of the skin and muscle, and has been described at high frequency in the Shetland sheepdog and Rough collie. Genetic investigations of this condition in the Shetland sheepdog have revealed an associated linkage region on canine chromosome 35 [9], but fine-mapping studies have been lacking. Additionally, a microarray study in the Shetland sheepdog was able to identify 285 genes, many with immune function, that were differentially regulated in cases vs controls [10]. In a study of familial dermatomyositis in four litters of Rough collies and Rough collie-Labrador crossbreeds, there was an association of disease severity with the DLA class II allele DLA-DRB1*015 but no association with two DLA class I genes [11].

We have previously been able to show DLA class II associations with a range of complex immune-mediated disorders, such as hypothyroid disease and diabetes mellitus [12], [13]. Building upon this we present the findings of the first DLA association study of a canine polymyositis.

## Materials and Methods

### Samples

Canine saliva samples (Oragene® ANIMAL, DNA Genotek Inc., Canada) or blood in ethylenediaminetetraacetic acid (EDTA) (residual blood from a diagnostic test) were submitted to the UK DNA Archive for Companion Animals, University of Manchester. Samples were submitted by veterinary surgeons and owners. In total there were 212 samples included in the study (29 cases and 183 controls), with samples selected from the UK wherever possible. However, three cases originated from New Zealand, Australia and Canada. Six control samples originated from Hungary and four from the USA.

Diagnosis of polymyositis was assigned with varying degrees of confidence, based on the presence or absence of particular criteria, summarised in Table 1. As well as the identification of consistent clinical signs [3], 12 cases had definitive histopathological confirmation by muscle biopsy and one by study at post-mortem. The most common biopsy site was the temporal muscle. Where this was the case, to rule out masticatory muscle myositis (MMM), dogs were required to be 2 M antibody negative (antibody directed against specific masticatory muscle fibres) or have an additional muscle site biopsy. A further four cases were classified as “probable” and had biopsies where 2 M antibody screening was not performed/pending but MMM was ruled out based on the presence of dysphagia and sialorrhea, features not considered typical of MMM. In these 17 cases with biopsy, myasthenia gravis (MG) was ruled out through a mixture of negative acetylcholine receptor antibody (AChR) tests, masticatory muscle atrophy, and high creatine kinase levels (>1000U/L). The remaining 12 dogs were classified as “possible” cases where biopsy was not performed but MMM and MG were ruled out based on clinical signs of dysphagia/sialorrhea and masticatory muscle atrophy, respectively.

**Table 1:** The criteria used to assign cases to a diagnostic confidence grouping.

	Diagnostic confidence grouping		
Criterion	Definite	Probable	Possible
Muscle biopsy showing inflammatory pathology	Yes	Yes	No
2 M antibody negative or additional muscle site biopsy	Yes	No	No
AChR antibody negative, high CK or MM atrophy	Yes	Yes	Yes
Dysphagia/sialorrhea	Yes	Yes	Yes
**Number of dogs**	**13**	**4**	**12**

Family history of polymyositis and the degree of relatedness between dogs were ascertained from pedigrees and owner reporting. There were 93 first degree unaffected relatives, defined as dogs showing no clinical signs of the disease that had at least one affected offspring, parent or sibling. 44 second degree unaffected relatives, defined as dogs showing no clinical signs of the disease that had at least one affected half-sibling, grandparent, grandchild, aunt, uncle, niece or nephew. 46 control dogs were selected, defined as dogs showing no clinical signs of polymyositis (based on information from owners and clinicians) and no reported family history of polymyositis. The average age of onset in cases was 2.92 years (range 0.5–8 years), consistent with previous observations [1], [2]. Dogs in the control group had an average age of 6.92 years, ranging from 0.58–13.83 years, with 17 out of 46 dogs over the age of 8 years.

### DNA Extraction

DNA was extracted from blood using QIAamp® DNA Blood Midi Kit (Qiagen, Crawley, UK). DNA from the Oragene® ANIMAL saliva kit was extracted using the manufacturer’s standard protocol. DNA concentration was measured using a NanoDrop® ND-1000 spectrophotometer (Wilmington, USA) and diluted in water to 5 ng/µl for PCR.

### Typing of DLA Class II Loci

All dogs were characterised for three class II loci: DLA-DRB1, DLA-DQA1, and DLA-DQB1 using Sequence-Based Typing (SBT) of exon 2 of each of these genes.

PCR was performed in a 96-well plate format. A 25 µl reaction volume was used per well, which consisted of: 5 µl of genomic DNA (5 ng/µl), 2.5 µl of 10x PCR buffer (with added MgCl_2_, 20 mM) (Roche), 0.5 µl of dNTPs (10 mM) (Roche), 0.5 µl of each forward and reverse primer, 0.13 µl FastStart Taq (5 U/µl) (Roche), and 15.87 µl of distilled water.

DRB1 primers: **DRBIn1** CCG TCC CCA CAG CAC ATT TC, **DRBIn2T7** TAA TAC GAC TCA CTA TAG GG TGT GTC ACA CAC CTC AGC ACC A. DQA1 primers: **DQAIn1** TAA GGT TCT TTT CTC CCT CT, **DQAIn2** GGA CAG ATT CAG TGA AGA GA. DQB1 primers: **DQB1BT7** TAA TAC GAC TCA CTA TAG GG CTC ACT GGC CCG GCT GTC TC, **DQBR2** CAC CTC GCC GCT GCA ACG TG. All primers are intronic and locus specific. The T7-tailed portion is underlined.

Amplification was performed using a touchdown PCR protocol on a PTC-225 tetrad cycler as follows: 95°C for 5 minutes; 14 touchdown cycles of: (95°C for 30 seconds, annealing for 1 minute starting at 62°C (DRB1) 54°C (DQA1) 73°C (DQB1) reducing by 0.5°C for each cycle, 72°C for 1 minute); 25 cycles of: (95°C for 1 minute, 55°C (DRB1) 47°C (DQA1) 66°C (DQB1) for 1 minute, 72°C for 1 minute); followed by a final elongation step of 72°C for 10 minutes. A negative control containing no DNA was included in each amplification to detect any contamination.

The presence of PCR product was assessed by running 5 µl of each reaction with 3 µl of loading buffer on a 2% agarose gel. Purification was performed by adding 4 µl of a 1in8 water dilution of USB® ExoSAP-IT® (Affymetrix, High Wycombe, UK) to 1 µl of PCR product and thermocycling at 37°C for 1 hour followed by 80°C for 15 minutes.

Sequencing (standard Sanger protocol) was performed in only one direction: reverse for DLA-DRB1 and DLA-DQA1 and forward for DLA-DQB1. The T7 primers (DRB1 and DQB1) or PCR primers (DQA1, DQAIn2) were used for sequencing. The product sizes are 303 bp for DLA-DRB1, 345 bp for DLA-DQA1 and 300 bp for DLA-DQB1. Sequencing data was analysed by *SBTengine*® (GenDx, Genome Diagnostics B.V., Utrecht, Netherlands). Briefly, the sequences were aligned to a consensus sequence and each polymorphic site was analysed either by the software or manually. The corrected sequence was then matched to a reference sequence library built into the software package (taken from http://www.ebi.ac.uk/ipd/mhc/dla/index.html).

Haplotypes were identified by first selecting the dogs that were homozygous for all three loci DLA-DRB1/DQA1/DQB1. This identified several different combinations of alleles. Secondly, dogs that were homozygous for only two of the loci were selected and the previous haplotypes were confirmed and added to. The remaining dogs were examined using the haplotype information already gathered.

### Statistical Analysis

Contingency tables (2×2) were used to calculate odds ratios, 95% confidence intervals (Cornfield) and p-values for each haplotype and allele. For the Chi-squared test (Χ^2^), Yates’s correction for continuity was utilised.

### Ethics Statement

Collection of veterinary blood samples solely for research purposes, without a home office licence, is prohibited in the United Kingdom. However, residual blood remaining after a diagnostic test may be used for research and does not require a licence. Collecting saliva with swabs is considered a non-invasive procedure and does not require a licence. Residual blood was submitted by the attending veterinary surgeon with owner permission. Saliva was collected and submitted by the owners of the animals.

## Results

DLA alleles and haplotypes were assigned to all dogs: affected dogs, unaffected first degree relatives of an affected dog, unaffected second degree relatives of an affected dog, and control dogs with no known family history of polymyositis. Within this group of Hungarian Viszlas, we identified seven haplotypes with a genotype count greater than five, plus four other less frequent haplotypes. Table 2 shows the frequencies of these haplotypes in each of the groups.

We did not identify any evidence of sex bias from our data but the small numbers make it difficult to draw definitive conclusions. In cases, there were marginally more males than females (Female: 41%, Male: 59%) while in the “graded controls” group there was a larger proportion of females compared to males (Female: 60%, Male: 40% - two dogs of unknown sex).

**Figure 1:**
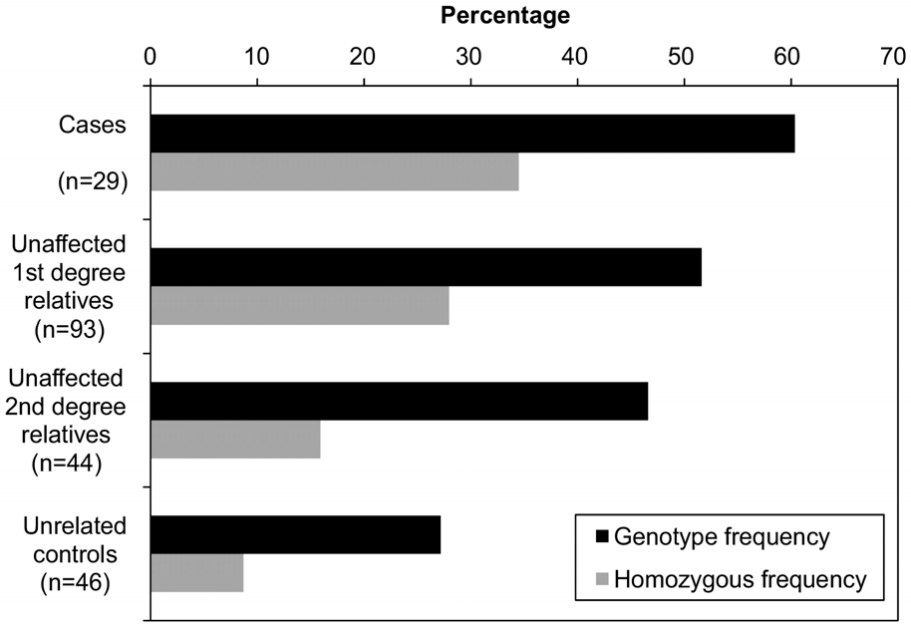
Haplotype and homozygous frequency of DLA-DRB1*02001/DQA1*00401/DQB1*01303 in cases and “graded controls”.

The most frequent haplotypes were DLA-DRB1*02001/DQA1*00401/DQB1*01303 and DLA-DRB1*00601/DQA1*005011/DQB1*00701, with 71% and 36% of all dogs, respectively, having at least one copy of this haplotype. One interesting observation is the marked frequency gradient of the DLA-DRB1*02001/DQA1*00401/DQB1*01303 haplotype with the highest frequency (60%) occurring in cases compared to 52% in unaffected first degree relatives, to 47% in unaffected second degree relatives, and to 27% in unrelated controls. This can be seen more clearly in Figure 1.

**Table 2:** Frequencies of the DLA haplotypes found in Hungarian Vizslas.

							*Graded Controls*					
Haplotype			Cases		All Controls		1st degree relatives		2nd degree relatives		Controls	
			*2n = 58*		*2n = 366*		*2n = 186*		*2n = 88*		*2n = 92*	
DRB1	DQA1	DQB1	n	%	n	%	n	%	n	%	n	%
02001	00401	01303	35	60.3	162	44.3	96	51.6	41	46.6	25	27.2
00601	005011	00701	12	20.7	72	19.7	35	18.8	14	15.9	23	25.0
00901	00101	008011	5	8.6	31	8.5	19	10.2	7	8.0	5	5.4
00801	00301	00401	3	5.2	44	12.0	17	9.1	12	13.6	15	16.3
02301	00301	00501	2	3.4	25	6.8	13	7.0	10	11.4	2	2.2
01501	00601	02301	1	1.7	16	4.4	5	2.7	2	2.3	9	9.8
04801	00101	008011	0	0	9	2.5	1	0.5	2	2.3	6	6.5
Other haplotypes			0	0	7	1.9	0	0	0	0	7	7.6

Comparison of cases against all dogs without polymyositis (including those dogs with a family history of affected animals) (n = 29 cases/183 controls), revealed a significant association of the DLA-DRB1*02001/DQA1*00401/DQB1*01303 haplotype with disease risk (Χ^2^ = 4.58, OR = 1.92, 95% CI 1.05–3.5, p = 0.032). As Hungarian Vizslas are recognised as being a breed predisposed to polymyositis, we further analysed the data to ascertain whether a stronger association could be observed for this haplotype if only dogs with no family history of the disease were considered. Thus, we removed dogs that had a family history of the disease, and compared the case and control groups alone (n = 29 cases/46 controls). In this analysis, the genotypic frequency in affected dogs (60%) was over twice that in control dogs (27%) (Χ^2^ = 15, OR = 4.08, 95% CI 1.92–8.74, p = 0.00011).

Given the differing levels of confidence in diagnosis amongst the 29 cases (Table 1), subgroup analyses were also conducted. In the 17 dogs with a “definite” or “probable” diagnosis, the genotypic frequency of DLA-DRB1*02001/DQA1*00401/DQB1*01303 was 64.7%. In the remaining 12 cases with a “possible” diagnosis, the frequency was 54.2%. Both of these frequencies were significantly different to the 27% frequency found in the group of 46 unrelated controls. For the “definite/probable” group this gave OR = 4.91, p = 0.00025, and for the “possible” group OR = 3.17, p = 0.024. Additionally the frequency of dogs homozygous for the risk haplotype in each subgroup was 35.3% and 33.3%, respectively.

As the age of onset in cases ranged up to 8 years, we assessed the frequency of the risk haplotype in a subset of 17 controls over the age of 8 years. This revealed a genotype frequency of 29.4%, which was significantly different compared to the frequency in affected dogs (OR 3.65, 95% CI 1.35–10.04, p = 0.0081).

Over a third of affected dogs were homozygous for DLA-DRB1*02001/DQA1*00401/DQB1*01303 (34.5%) compared to 8.7% of control dogs. Table 3 shows that when comparing genotypes, using a baseline of dogs that did not carry any copies of the risk haplotype, there is a significant effect on disease risk with heterozygote presence of the haplotype (OR = 5) and the effect is increased substantially for homozygotes (OR = 15). Homozygosity of the risk haplotype did not appear to reduce the age of onset for affected dogs; the average age of onset for dogs homozygous for the risk haplotype was 3.28 years compared to 2.83 years for other dogs.

**Table 3:** Association of the DLA-DRB1*02001/DQA1*00401/DQB1*01303 genotypes with polymyositis, relative to the disease risk in individuals who are DLA-DRB1*02001/DQA1*00401/DQB1*01303 homozygous negative.

Genotype	Cases	Controls	OR	95% CI
	*n* = 29	*n* = 46	(compared to controls)	
DRB1/DQA1/DQB1	*n (%)*	*n (%)*		
02001/00401/01303 (−/−)	4 (13.8)	24 (52.2)	1	Baseline
02001/00401/01303 (−/+)	15 (51.7)	18 (39.1)	5	1.25–21.67
02001/00401/01303 (+/+)	10 (34.5)	4 (8.7)	15	2.54–104.45

There is some indication that the presence of DLA-DRB1*01501/DQA1*00601/DQB1*02301 is protective, with 9/46 unrelated control dogs having one copy compared to only 1/29 cases. However, this may be a reflection of the reduction in non-disease associated haplotypes in cases due the high frequency of the DLA-DRB1*02001/DQA1*00401/DQB1*01303 haplotype.

As there were two major haplotypes that carried DQA1*00101 and DQA1*00301, an allelic association analysis for each locus was also performed. This showed no significant differences between cases and controls carrying either allele (data not shown).

## Discussion

This study provides evidence for an association between an MHC class II haplotype with the development of polymyositis in Hungarian Vizslas. In the dog population as a whole, the DLA-DRB1*02001/DQA1*00401/DQB1*01303 haplotype is relatively common with a haplotype frequency of 6.2% amongst approximately 10,000 dogs from over 200 breeds tested (LJ Kennedy, unpublished data). The limited DLA class II haplotype diversity of the Hungarian Vizsla is typical of many other breeds studied [14], including the Pug dog [15].

The DLA-DRB1*02001 allele does not match the DLA-DRB1*015 allele previously associated with increased disease risk in canine dermatomyositis [11], which supports a divergent aetiology for the two diseases. As there are other known canine inflammatory myopathies, it will be important to investigate if other MHC associations exist in these diseases and whether they share the same risk DLA haplotype as presented here. Given the previous observation of CD4+ T-cells being found in excess CD8+ T-cells in Hungarian Vizsla polymyositis biopsies, it would be particularly interesting to see if there are comparisons with canine masticatory muscle myositis, a disease where this is also a feature [1]. Furthermore, although MHC class I associations were not identified in a canine dermatomyositis [11], they have been demonstrated in human inflammatory myopathies, so it will be interesting to look for such an association in the canine conditions.

The DLA-DRB1*02001/DQA1*00401/DQB1*01303 haplotype has not previously been identified as being risk-associated in an immune-mediated disease. However, Wilbe et al. [16] found the haplotype to be protective against the immune-mediated condition of Symmetrical Lupoid Onychodystrophy (SLO) in Gordon Setters.

Carrying a single copy of the DLA-DRB1*02001/DQA1*00401/DQB1*01303 risk haplotype increases risk of polymyositis. Homozygosity of the risk haplotype further increases this risk. A possible explanation for this is that signalling through the MHC receptor requires a high threshold before a response is induced. Doubling the number of identical MHC molecules would allow this level to be reached more easily and possibly initiate autoimmunity to self-antigen. An alternative explanation could be that other susceptibility genes within the DLA region and beyond could be present on an extended haplotype [17]. This type of gene dosage effect has been shown with MHC associations in human autoimmune diseases, such as the presence of the shared epitope in rheumatoid arthritis [18] and HLA-DR2 in multiple sclerosis [17]. Homozygosity of MHC risk alleles has also been associated with increased disease risk in a canine SLE-related condition [19] and canine necrotizing meningoencephalitis [15]. It has also been associated with earlier disease onset in canine anal furunculosis [20], but this was not observed with polymyositis in this study.

The DLA-DRB1*02001/DQA1*00401/DQB1*01303 haplotype is more prevalent within pedigrees of affected Hungarian Vizsla cases, with a marked trend based on the degree of relatedness to an individual with the disease. This suggests that this haplotype has a low penetrance and provides evidence of a true association of MHC class II with polymyositis in Hungarian Vizslas; low penetrance of MHC alleles and haplotypes is expected and is a common feature in both canine and human MHC class II association studies [21]. However, this statement is not always correct and there are examples of highly penetrant MHC associations, as in the study of Pug dogs with necrotizing meningoencephalitis [15]. Although we stratified dogs based on the degree of family history of disease, the pedigree data for all controls were incomplete. Thus we were unable to model this penetrance.

Given the increased risk of polymyositis with homozygosity of the risk haplotype, it is tempting to speculate that selecting against this haplotype in the population would reduce the occurrence of the disease. However, this would reduce the diversity of an already restricted DLA repertoire and could have unintended negative consequences, such as an increased susceptibility to particular pathogens. It is also likely that polymyositis in this breed is a complex disorder, in which DLA class II genes are only part of a wider array of susceptibility loci.

The use of cases and controls from areas of the world other than the UK did not reveal any evidence of heterogeneity and thus we included the small number of these animals in the analysis. Interestingly, one haplotype was found only in three of the four control dogs from the USA, which could indicate some minor level of population stratification. However, the removal of the four samples from the USA did not change the overall results (data not shown). Given that this study was conducted within a single breed, with the majority of those dogs from UK lineages, the effects are unlikely to be due to population stratification.

Despite the differing levels of confidence in diagnosis of polymyositis in this study, we were able to show significant associations of the risk haplotype in all subgroups. However, future work will focus on improving the diagnosis of polymyositis in those dogs classified as “probable” or “possible” cases.

In conclusion, this study supports the hypothesis of an immune-mediated aetiology for this breed-specific polymyositis in the Hungarian Vizsla. The low penetrance of the risk haplotype indicates that other genetic and environmental factors will be involved. Although there are differences between the canine and human disease, mainly regarding different cellular infiltrates, the Hungarian Vizsla condition could present an important model for comparative studies. The rarity of polymyositis in humans (5–10 cases per million adults per year) [22] makes genetic association studies difficult due the problem of accruing sufficient sample sizes to gain adequate statistical power and provide replication cohorts. Pedigree dog breeds that display high predisposition for certain spontaneously occurring diseases have already proved to represent a powerful comparative genetic model for aiding in the discovery of novel genetic loci underlying analogous human conditions [23]. Reduced genetic heterogeneity and increased long-range linkage disequilibrium (LD) make the dog particularly amenable to genome-wide association studies (GWAS), with fewer individuals than would be required for an equivalent study in humans [24]. A future canine GWAS in Hungarian Vizslas could be an efficient and practical way of advancing our understanding of both the canine and human conditions of polymyositis and indeed, other inflammatory disorders.
